# Role of Inflammatory Processes in the Brain-Body Relationship Underlying Hypertension

**DOI:** 10.1007/s11906-023-01268-y

**Published:** 2023-10-03

**Authors:** Daniela Carnevale

**Affiliations:** 1 Department of Angiocardioneurology and Translational Medicine, IRCCS Neuromed, 86077 Pozzilli, IS Italy; 2 Department of Molecular Medicine, Sapienza University of Rome, 00161 Rome, Italy

**Keywords:** Hypertension, Neuroinflammation, Peripheral immune response, Autonomic nervous system, Cytokines

## Abstract

**Purpose of Review:**

Essential hypertension is a huge health problem that significantly impacts worldwide population in terms of morbidity and mortality. Idiopathic in its nature, elevated blood pressure results from a complex interaction between polygenic components and environmental and lifestyle factors. The constant growth in the burden of hypertension is at odds with expectations, considering the availability of therapeutic strategies. Hence, there is an endless need to further investigate the complexity of factors contributing to blood pressure elevation.

**Recent Findings:**

Recent data indicate that bidirectional interactions between the nervous system and the immune system alter inflammation in the brain and periphery, contributing to chronic hypertension. These findings indicate that the nervous system is both a direct driver of hypertension and also a target of feedback that often elevates blood pressure further. Similarly, the immune system is both target and driver of the blood pressure increases. The contributions of the feedback loops among these systems appear to play an important role in hypertension.

**Summary:**

Together, recent mechanistic studies strongly suggest that the interactions among the brain, immune system, and inflammation affect the participation of each system in the pathogenesis of hypertension, and thus, all of these systems must be considered in concert to gain a full appreciation of the development and potential treatments of hypertension.

## Introduction

Hypertension is an epidemic health challenge recognized as major worldwide risk factor for the rising incidence of cardiovascular disease [1]. Numerous antihypertensive drugs are available to tailor therapies, yet many patients fail to stay in an optimal range of blood pressure control, contributing to the increasing prevalence of uncontrolled hypertension [2].

The neurogenic basis of hypertension is usually ascribed to a sustained overactivation of the sympathetic nervous system (SNS) [3–5]. The persistent SNS stimulation of the heart, vasculature, and kidneys consequently affects cardiac output, vascular resistance, and fluid retention, overall contributing to blood pressure elevation [3]. On another note, the spectrum of factors contributing to etiology of hypertension has been widened by the observation that inflammation is involved in the onset of elevated blood pressure [6]. A multitude of studies conducted in the past decade demonstrated that activated T lymphocytes and macrophages accumulate in the kidneys and peripheral vasculature of experimental models of hypertension, likely contributing to blood pressure increase and end organ damage (see [7] for review). The SNS is also a main physiological system that provides a functional interface between the nervous and the immune systems. Hence, it has been postulated a new role of the autonomic control in hypertension, partly attributed to the modulation of immune responses [8, 9•, 10]. The autonomic nervous system comprises fibers that innervate organs of the immune system to modulate their functions through neurotransmitters’ release. Besides expressing receptors for neurotransmitters, immune cells also respond to the hypothalamic pituitary adrenal (HPA) axis by binding glucocorticoids, hence indicating that SNS and HPA axis represent a further level of control of innate and adaptive immune responses [9•]. Additionally, the adipose tissue that surrounds the vasculature is directly entangled by SNS fibers and provides a fine regulation of metabolic and endocrine functions contributing to blood pressure regulation [11]. Since perivascular adipose tissue can also directly release noradrenaline independently from innervation, it has been postulated that another route of cardiovascular control is ascribable to neuroimmune interactions established at a local vascular level [12, 13].

The autonomic nervous system utilizes efferent neural circuits, dependent on specific forebrain and hindbrain areas, to exert a direct control of peripheral tissues. Among the brain areas involved, the circumventricular organs (CVOs), the paraventricular nucleus of the hypothalamus (PVN), the rostral ventral lateral medulla (RVLM), the anteroventral third ventricle (AV3V), and the nucleus of the solitary tract (NTS) are well known to participate in the brain-mediated cardiovascular control [14–16]. Notably, a common property of the above-mentioned brain structures is the high expression of Angiotensin II type 1 receptors (AT1R), making them sensitive to Angiotensin II signaling [17–20]. CVOs are characterized by an elevated permeability to circulating substances and by the presence of fenestrated capillaries, enabling them to regulate bidirectional exchanges of hormones and soluble substances that usually do not cross the intact blood–brain barrier (BBB) [21].

Cytokines play an additional role in the bidirectional brain-body communication. In fact, the entire spectrum of cells composing the nervous system, ranging from neurons to glial and immune cells as well as to vascular cells, is endowed with cytokines’ receptors, enabling them to respond challenges coming from the periphery. Cytokines produced in response to tissues’ perturbation cross the BBB at sites of increased permeability, providing feedback to the brain. Sensitivity to peripheral disturbances depends on the ability of the brain to respond to the stimulation exerted by pro-inflammatory cytokines, like tumor necrosis factor-alpha (TNF-α) and interleukin-1 beta (IL-1β), which contribute to enhancement of sympathetic outflow and subsequent BP elevation [22, 23]. With ongoing neuroinflammation, glial cells and neurons contribute to cytokine release in the brain itself to further signal back to the peripheral tissues. This neuroinflammatory brain-body circuit is an additional mechanism involved in onset of hypertension [21]. Cytokines like IL-1β, TNF-a, and interleukin-6 (IL-6) might also directly impact the cerebral vasculature altering adherent and tight junctions of capillaries, further increasing BBB permeabilization and brain injury [24, 25].

Peripherally produced Angiotensin II is an additional signal that contributes to the brain-body bidirectional communication. Circulating Angiotensin II can cross the BBB to activate neuronal circuits within areas characterized by increased permeability [26]. On the other hand, Angiotensin II contributes to BBB disruption [27], further facilitating permeability in brain areas like the PVN, RVLM, and NTS, which are usually inaccessible because lined by a tight BBB [28].

The above observations have delineated a brain-body feedback and feedforward mechanism that recognizes brain inflammation as a cause/effect process involved in the etiology of hypertension.

## Can Neuroinflammation Trigger Blood Pressure Elevation?

Peripheral sympathoexcitation is mostly related to the stimulation of AT1R within the PVN [17–19, 29], whereby its persistent activation has been frequently recognized as a major cause of neurogenic hypertension. Notably, chronic infusion of Angiotensin II, which is a well-validated rodent model of human hypertension, activates the inflammatory signaling mediated by nuclear factor kappa B (NFkB) in the PVN [30], further sustaining neuroinflammation. At the same time, pro-inflammatory cytokines like IL-1β, directly administered into the PVN or infused intracerebroventricularly, determine an elevation of mean blood pressure levels [30–32].

The chronic administration of Angiotensin II also increases leukocyte adhesion to brain capillaries and venules, with consequent rolling and infiltration in the brain parenchyma [21]. At the cellular level, it has been observed that the process of leukocyte adhesion takes place concomitantly to the development of oxidative stress, and both phenomena precede blood pressure increase [27]. An additional tract that characterizes the process of brain inflammation ensuing upon Angiotensin II infusion is increased BBB permeability, which in turn facilitates leakage of the circulating vasoactive peptide in the PVN, RVLM, and NTS [28, 33•]. Studies that have utilized a treatment with tempol, a superoxide dismutase mimetic, showed a rescue of cerebrovascular inflammation and a restoration of BBB function [27], suggesting the dependence of this process on neuroinflammation-induced vascular oxidative stress.

Prorenin and its receptor (prorenin receptor, PRR)—additional members of the renin-angiotensin system (RAS)—can contribute to neurogenic hypertension by eliciting neuroinflammatory processes [34–36]. In fact, prorenin activates NFkB signaling in the NTS and enhances the expression of the pro-inflammatory cytokines IL-1b and TNF-α [37]. In microglia, prorenin has the further ability to enhance Angiotensin II effects, eliciting a heightened cytokine production.

Evidence that prorenin contributes to neurogenic-mediated blood pressure elevation has been provided in additional models of experimental hypertension. As an example, the downregulation of PRR in the supraoptic nucleus of the spontaneously hypertensive rat (SHR), obtained by viral transfection, effectively counteracted hypertension [38]. Also, inhibition of PRR in the brain rescued blood pressure elevation and sympathetic outflow in double transgenic hypertensive mice expressing human renin-angiotensinogen [39]. The selective deletion of PRR in neurons of mice subjected to hypertension induced by deoxycorticosterone acetate (DOCA)-salt counteracted Angiotensin II production and blood pressure elevation [39, 40].

On the other hand, strategies hampering the inflammatory burden proved effective in ameliorating blood pressure elevation. Dampening inflammation by administration of interleukin-10 (IL-10) or minocycline successfully reduced microglial reactivity and blood pressure increase [41]. Additionally, minocycline hindered the increase in pro-inflammatory cytokines and the concomitant decrease in IL-10 induced by Angiotensin II on production [31, 41, 42].

The PVN is not the sole brain region contributing to neurogenic hypertension. Other studies executed in the SHR imply that an inflammatory state exists in the NTS too [43]. Also, a relationship among RVLM cytokines’ expression and burden of oxidative stress, enhanced SNS outflow, and hypertensive responses has been proposed. Moreover, it has been demonstrated that the signaling of angiotensin-converting enzyme in the PVN induces pro-inflammatory cytokines in the RVLM through direct projections of the PVN, resulting in sympathoexcitation and oxidative stress [40].

Taken together, the studies here recapitulated show that RAS activation and consequent hypertension result from a mixed effect of feedforward signaling of Angiotensin II and prorenin on the PVN and RVLM and feedback effect of circulating Angiotensin II and cytokines acting on the CVOs.

## Can Peripheral Inflammatory Conditions Trigger Feedforward Neurogenic Hypertension?

Manifold processes activate a state of inflammation in the peripheral tissues and, depending on the causes and pathophysiological context, feedback signals to the brain can be evoked to elicit reflex responses that might help to restore the homeostasis. At the same time, the activation of specific brain areas sensitive to pro-inflammatory signaling can in turn recruit pro-hypertensive responses. Hence, peripheral inflammatory conditions might provide an enhancement of ongoing neurogenic hypertension or trigger blood pressure elevation per se. This part of the review will encompass the studies that describe some of the common conditions leading to persistent peripheral inflammation and recognized as factors predisposing to hypertension. Among these, it is noteworthy mentioning hyperlipidic diets, autoimmune disease, and commensal bacteria dysbiosis.

A typical chronic low-grade inflammation is induced by obesity, often accompanied by increased RAS activity [44]. High-fat diet (HFD)-induced obesity triggers microglial reactivity in specific brain areas [45]. In particular, animals fed with HFD are characterized by neuroinflammatory processes involved in BP and metabolism regulation, established in the PVN and the SFO. Notably, these areas are also sensitive to Angiotensin II, and on the other hand, diet and leptin have been shown to enhance AT1aR expression in glial cells and neurons [35, 44], suggesting the existence of mutual interactions between RAS and neuroinflammation during metabolic derangement. A further relationship with peripheral increase of autonomic activity has been identified. Mice with an inhibition of AT1aR signaling in the brain display an attenuated response to leptin in terms of sympathetic outflow to key districts involved in cardiovascular regulation, namely, renal and brown adipose tissues [35]. However, no effect on metabolic phenotypes and feeding behavior was observed. Other works have demonstrated that leptin sensitizes key brain areas, namely, the NTS [46], the SFO [47], and the RVLM [48], to affect the cardiovascular responses mediated by RAS neurohumoral control.

Immune factors exert actions in the brain that exacerbate hypertension. Notably, experimental models of hypertension display enhanced activation of immune reactions in the brain, whereas neuroinflammatory processes trigger blood pressure increase [49], hence suggesting that there is a bidirectional regulation between brain and peripheral inflammatory processes and blood pressure regulation.

Autoimmune diseases are correlated with heightened cardiovascular risk, yet the mechanistic contribution of immune derangement to cardiovascular pathophysiology is still object of investigation. What is well known is the consistent association reported between hypertension and autoimmune disorders. Also, hypertension in this specific population of patients is characterized by earlier manifestation and higher resistance to treatment. Autoimmune conditions more frequently associated with enhanced cardiovascular risk are systemic lupus erythematosus, psoriasis, rheumatoid arthritis, and systemic sclerosis.

Patients affected by systemic lupus erythematosus have an elevated risk of developing hypertension, which overall contributes to most of the cardiovascular-related morbidity and mortality in this category of individuals [50]. A key feature of systemic lupus erythematosus is the formation of immune complexes composed by autoantibodies that deposit in various tissues, especially in kidneys. As a result, glomerulonephritis ensues in a vast majority of patients, resulting in renal dysfunction [51]. Nonetheless, data supporting a direct relationship between the onset and progression of glomerulonephritis and hypertension are missing, suggesting the further studies will be necessary to clarify the underlying mechanisms.

Similarly, patients suffering from psoriasis have high prevalence of hypertension, which typically correlates with disease severity [52]. This risk is particularly high in the subset of patients with psoriatic arthritis. Since a positive correlation has been found between psoriasis and metabolic syndrome [53], it has been postulated that the relationship with hypertension might be partly dependent on this aspect. However, no definitive data is available.

Rheumatoid arthritis is a further autoimmune disorder typically associated with enhanced cardiovascular risk [54]. While no clear mechanistic information is available to support this relationship, it is well recognized that the variations in the incidence of hypertension depend on confounding factors, like age, ethnicity, and therapeutic compliance.

On a different note, it is less clear whether other diseases related to a derangement of immune system, namely, systemic sclerosis, have a pathophysiological relationship with hypertension [55]. Key tracts of systemic sclerosis that might favor the hypothesis of being considered a triggering condition for the development of hypertension are related to the frequently reported vascular endothelial dysfunction manifested by these patients [56]. Also, scleroderma, a typical feature of systemic sclerosis, leads to hypoxic mechanisms in vascular tissues, lastly resulting in fibrosis and dysfunction. The resulting chronic vascular inflammation might represent a key contributor to progression of vascular fibrosis, impairment of vascular function and cardiovascular disease progression.

Peripheral inflammatory and immune homeostasis might be also challenged by conditions that alter a proper commensal relationship with bacteria, particularly those of the gut and oral microbiome. In fact, periodontitis and gut dysbiosis are associated with an increased risk of cardiovascular disease, particularly hypertension. Periodontitis results in a local autoimmune process that affects the cardiovascular system and risk of hypertension [57, 58•]. Other cardiovascular conditions that associate, in combination with hypertension or alone, with periodontitis are represented by renal disease, left ventricular hypertrophy, and atherosclerosis [59, 60]. A similarly increased risk of cardiovascular disease, particularly hypertension, is observed in patients suffering from gut dysbiosis [61]. The capability of specific metabolites produced by altered gut microbiota to trigger the autonomic nervous system has been postulated to represent a main contributor to neurogenic hypertension [62, 63]. Additional mechanisms have been also identified, discovering that chemosensing receptors, like the olfactory receptors, expressed in the juxtaglomerular apparatus of kidneys respond to short chain fatty acids by activating renin secretion and hence contributing to blood pressure dysregulation [64].

## Conclusions and Perspectives

**Fig. 1:**
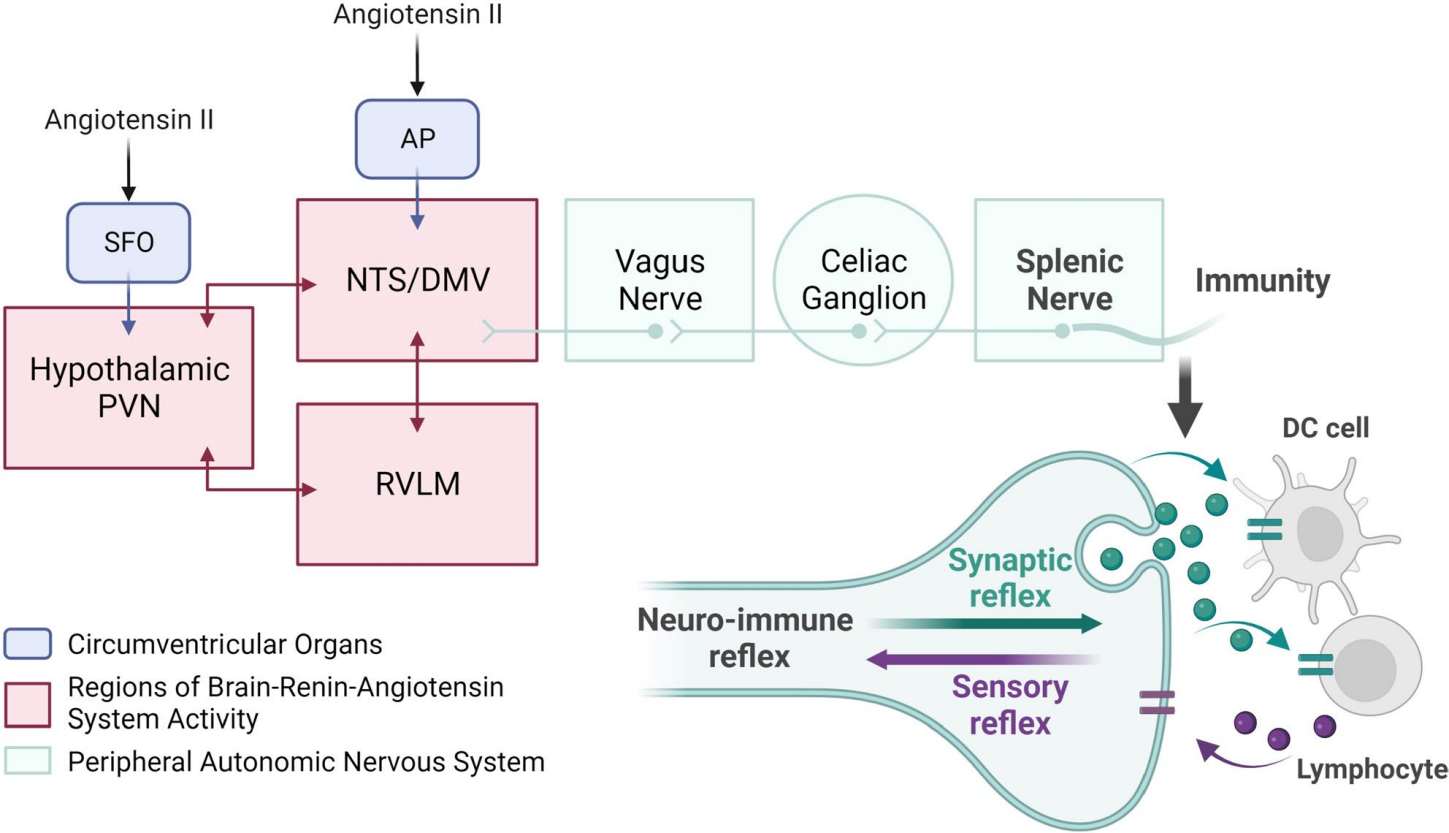
Schematics of the bidirectional communication established between the nervous and immune system. Brain areas sensitive to circulating substances like Angiotensin II are represented in blue boxes. Key brain areas composing the brain renin angiotensin system are represented by the red boxes. Green boxes and circles represent the peripheral efferent of the neuroimmune communication involved in cardiovascular diseases. Abbreviations: subfornical organ (SFO); area postrema (AP); paraventricular nucleus of the hypothalamus (PVN); nucleus of the solitary tract (NTS)/dorsal motor nucleus of the vagus nerve (DMV); rostroventrolateral medulla (RVLM)

Chronic inflammation and immune dysregulation are important comorbidities in hypertension. In fact, the treatment of inflammation improves blood pressure control, ameliorating cardiovascular function and reducing the overall cardiovascular risk. Together, the studies summarized in this review provide support to the concept that the brain is central in the interactions established between inflammatory and immune dysregulation and blood pressure regulation. Revealing the underlying mechanisms might unveil promising avenues for new therapeutics in neurogenic hypertension (Fig. 1).

## References

[1] Kearney PM, Whelton M, Reynolds K, Muntner P, Whelton PK, He J (2005). Global burden of hypertension: analysis of worldwide data. Lancet.

[2] Chobanian AV. Shattuck Lecture. The hypertension paradox--more uncontrolled disease despite improved therapy. N Engl J Med. 2009;361(9):878–87. 10.1056/NEJMsa0903829. 10.1056/NEJMsa0903829 19710486

[3] Esler M (2015). The sympathetic nervous system in hypertension: back to the future?. Curr Hypertens Rep.

[4] Takahashi H (2012). Upregulation of the Renin-Angiotensin-aldosterone-ouabain system in the brain is the core mechanism in the genesis of all types of hypertension. Int J Hypertens.

[5] Karim S, Chahal A, Khanji MY, Petersen SE, Somers VK (2023). Autonomic cardiovascular control in health and disease. Compr Physiol.

[6] Coffman TM (2011). Under pressure: the search for the essential mechanisms of hypertension. Nat Med.

[7] Drummond GR, Vinh A, Guzik TJ, Sobey CG (2019). Immune mechanisms of hypertension. Nat Rev Immunol.

[8] Esler M (2011). The sympathetic nervous system through the ages: from Thomas Willis to resistant hypertension. Exp Physiol.

[9] Carnevale D (2022). Neuroimmune axis of cardiovascular control: mechanisms and therapeutic implications. Nat Rev Cardiol.

[10] Elenkov IJ, Wilder RL, Chrousos GP, Vizi ES (2000). The sympathetic nerve–an integrative interface between two supersystems: the brain and the immune system. Pharmacol Rev.

[11] Fliers E, Kreier F, Voshol PJ, Havekes LM, Sauerwein HP, Kalsbeek A (2003). White adipose tissue: getting nervous. J Neuroendocrinol.

[12] Ayala-Lopez N, Martini M, Jackson WF, Darios E, Burnett R, Seitz B (2014). Perivascular adipose tissue contains functional catecholamines. Pharmacol Res Perspect.

[13] Sharma M, Schlegel M, Brown EJ, Sansbury BE, Weinstock A, Afonso MS et al. Netrin-1 alters adipose tissue macrophage fate and function in obesity. Immunometabolism. 2019;1(2). 10.20900/immunometab20190010. 10.20900/immunometab20190010 PMC6699780 31428465

[14] Szczepanska-Sadowska E, Cudnoch-Jedrzejewska A, Ufnal M, Zera T (2010). Brain and cardiovascular diseases: common neurogenic background of cardiovascular, metabolic and inflammatory diseases. J Physiol Pharmacol.

[15] Kasparov S, Teschemacher AG (2008). Altered central catecholaminergic transmission and cardiovascular disease. Exp Physiol.

[16] Xi H, Li X, Zhou Y, Sun Y. The regulatory effect of the paraventricular nucleus on hypertension. neuroendocrinology. 2023. 10.1159/000533691. 10.1159/000533691 37598678

[17] Elsaafien K, de Kloet AD, Krause EG, Sumners C (2020). Brain angiotensin type-1 and type-2 receptors in physiological and hypertensive conditions: focus on neuroinflammation. Curr Hypertens Rep.

[18] Frazier CJ, Harden SW, Alleyne AR, Mohammed M, Sheng W, Smith JA (2021). An angiotensin-responsive connection from the lamina terminalis to the paraventricular nucleus of the hypothalamus evokes vasopressin secretion to increase blood pressure in mice. J Neurosci.

[19] Wang L, Hiller H, Smith JA, de Kloet AD, Krause EG (2016). Angiotensin type 1a receptors in the paraventricular nucleus of the hypothalamus control cardiovascular reactivity and anxiety-like behavior in male mice. Physiol Genomics.

[20] Forrester SJ, Booz GW, Sigmund CD, Coffman TM, Kawai T, Rizzo V (2018). Angiotensin II signal transduction: an update on mechanisms of physiology and pathophysiology. Physiol Rev.

[21] Santisteban MM, Iadecola C, Carnevale D (2023). Hypertension, neurovascular dysfunction, and cognitive impairment. Hypertension.

[22] Wei SG, Yu Y, Felder RB (2018). Blood-borne interleukin-1beta acts on the subfornical organ to upregulate the sympathoexcitatory milieu of the hypothalamic paraventricular nucleus. Am J Physiol Regul Integr Comp Physiol.

[23] Wei SG, Zhang ZH, Beltz TG, Yu Y, Johnson AK, Felder RB (2013). Subfornical organ mediates sympathetic and hemodynamic responses to blood-borne proinflammatory cytokines. Hypertension.

[24] Labus J, Hackel S, Lucka L, Danker K (2014). Interleukin-1beta induces an inflammatory response and the breakdown of the endothelial cell layer in an improved human THBMEC-based in vitro blood-brain barrier model. J Neurosci Methods.

[25] Rochfort KD, Collins LE, Murphy RP, Cummins PM (2014). Downregulation of blood-brain barrier phenotype by proinflammatory cytokines involves NADPH oxidase-dependent ROS generation: consequences for interendothelial adherens and tight junctions. PLoS ONE.

[26] Paton JF, Wang S, Polson JW, Kasparov S (2008). Signalling across the blood brain barrier by angiotensin II: novel implications for neurogenic hypertension. J Mol Med (Berl).

[27] Zhang M, Mao Y, Ramirez SH, Tuma RF, Chabrashvili T (2010). Angiotensin II induced cerebral microvascular inflammation and increased blood-brain barrier permeability via oxidative stress. Neuroscience.

[28] Biancardi VC, Son SJ, Ahmadi S, Filosa JA, Stern JE (2014). Circulating angiotensin II gains access to the hypothalamus and brain stem during hypertension via breakdown of the blood-brain barrier. Hypertension.

[29] Paton JF, Raizada MK (2010). Neurogenic hypertension. Exp Physiol.

[30] Cardinale JP, Sriramula S, Mariappan N, Agarwal D, Francis J (2012). Angiotensin II-induced hypertension is modulated by nuclear factor-kappaBin the paraventricular nucleus. Hypertension.

[31] Kang YM, He RL, Yang LM, Qin DN, Guggilam A, Elks C (2009). Brain tumour necrosis factor-alpha modulates neurotransmitters in hypothalamic paraventricular nucleus in heart failure. Cardiovasc Res.

[32] Shi Z, Gan XB, Fan ZD, Zhang F, Zhou YB, Gao XY (2011). Inflammatory cytokines in paraventricular nucleus modulate sympathetic activity and cardiac sympathetic afferent reflex in rats. Acta Physiol (Oxf).

[33] Santisteban MM, Ahn SJ, Lane D, Faraco G, Garcia-Bonilla L, Racchumi G (2020). Endothelium-macrophage crosstalk mediates blood-brain barrier dysfunction in hypertension. Hypertension.

[34] Nguyen G, Muller DN (2010). The biology of the (pro)renin receptor. J Am Soc Nephrol.

[35] Hilzendeger AM, Morgan DA, Brooks L, Dellsperger D, Liu X, Grobe JL (2012). A brain leptin-renin angiotensin system interaction in the regulation of sympathetic nerve activity. Am J Physiol Heart Circ Physiol.

[36] Nakagawa P, Gomez J, Grobe JL, Sigmund CD (2020). The renin-angiotensin system in the central nervous system and its role in blood pressure regulation. Curr Hypertens Rep.

[37] Zubcevic J, Jun JY, Lamont G, Murca TM, Shi P, Yuan W (2013). Nucleus of the solitary tract (pro)renin receptor-mediated antihypertensive effect involves nuclear factor-kappaB-cytokine signaling in the spontaneously hypertensive rat. Hypertension.

[38] Shan Z, Shi P, Cuadra AE, Dong Y, Lamont GJ, Li Q (2010). Involvement of the brain (pro)renin receptor in cardiovascular homeostasis. Circ Res.

[39] Li W, Peng H, Cao T, Sato R, McDaniels SJ, Kobori H (2012). Brain-targeted (pro)renin receptor knockdown attenuates angiotensin II-dependent hypertension. Hypertension.

[40] Li W, Peng H, Mehaffey EP, Kimball CD, Grobe JL, van Gool JM (2014). Neuron-specific (pro)renin receptor knockout prevents the development of salt-sensitive hypertension. Hypertension.

[41] Shi P, Diez-Freire C, Jun JY, Qi Y, Katovich MJ, Li Q (2010). Brain microglial cytokines in neurogenic hypertension. Hypertension.

[42] Kang YM, Ma Y, Zheng JP, Elks C, Sriramula S, Yang ZM (2009). Brain nuclear factor-kappa B activation contributes to neurohumoral excitation in angiotensin II-induced hypertension. Cardiovasc Res.

[43] Waki H, Gouraud SS, Maeda M, Raizada MK, Paton JF (2011). Contributions of vascular inflammation in the brainstem for neurogenic hypertension. Respir Physiol Neurobiol.

[44] de Kloet AD, Pioquinto DJ, Nguyen D, Wang L, Smith JA, Hiller H (2014). Obesity induces neuroinflammation mediated by altered expression of the renin-angiotensin system in mouse forebrain nuclei. Physiol Behav.

[45] Thaler JP, Yi CX, Schur EA, Guyenet SJ, Hwang BH, Dietrich MO (2012). Obesity is associated with hypothalamic injury in rodents and humans. J Clin Invest.

[46] Mark AL, Agassandian K, Morgan DA, Liu X, Cassell MD, Rahmouni K (2009). Leptin signaling in the nucleus tractus solitarii increases sympathetic nerve activity to the kidney. Hypertension.

[47] Smith PM, Ferguson AV (2012). Cardiovascular actions of leptin in the subfornical organ are abolished by diet-induced obesity. J Neuroendocrinol.

[48] Marsh AJ, Fontes MA, Killinger S, Pawlak DB, Polson JW, Dampney RA (2003). Cardiovascular responses evoked by leptin acting on neurons in the ventromedial and dorsomedial hypothalamus. Hypertension.

[49] Wu KL, Chan SH, Chan JY (2012). Neuroinflammation and oxidative stress in rostral ventrolateral medulla contribute to neurogenic hypertension induced by systemic inflammation. J Neuroinflammation.

[50] Taylor EB, Ryan MJ (2016). Understanding mechanisms of hypertension in systemic lupus erythematosus. Ther Adv Cardiovasc Dis.

[51] Daniel L, Sichez H, Giorgi R, Dussol B, Figarella-Branger D, Pellissier JF (2001). Tubular lesions and tubular cell adhesion molecules for the prognosis of lupus nephritis. Kidney Int.

[52] Armstrong EJ, Harskamp CT, Armstrong AW (2013). Psoriasis and major adverse cardiovascular events: a systematic review and meta-analysis of observational studies. J Am Heart Assoc.

[53] Armstrong AW, Harskamp CT, Armstrong EJ (2013). Psoriasis and metabolic syndrome: a systematic review and meta-analysis of observational studies. J Am Acad Dermatol.

[54] del Rincon ID, Williams K, Stern MP, Freeman GL, Escalante A (2001). High incidence of cardiovascular events in a rheumatoid arthritis cohort not explained by traditional cardiac risk factors. Arthritis Rheum.

[55] Man A, Zhu Y, Zhang Y, Dubreuil M, Rho YH, Peloquin C (2013). The risk of cardiovascular disease in systemic sclerosis: a population-based cohort study. Ann Rheum Dis.

[56] Altorok N, Wang Y, Kahaleh B (2014). Endothelial dysfunction in systemic sclerosis. Curr Opin Rheumatol.

[57] Czesnikiewicz-Guzik M, Osmenda G, Siedlinski M, Nosalski R, Pelka P, Nowakowski D (2019). Causal association between periodontitis and hypertension: evidence from Mendelian randomization and a randomized controlled trial of non-surgical periodontal therapy. Eur Heart J.

[58] Munoz Aguilera E, Suvan J, Buti J, Czesnikiewicz-Guzik M, Barbosa Ribeiro A, Orlandi M (2020). Periodontitis is associated with hypertension: a systematic review and meta-analysis. Cardiovasc Res.

[59] Yang Q, Ding H, Wei W, Liu J, Wang J, Ren J (2021). Periodontitis aggravates kidney injury by upregulating STAT1 expression in a mouse model of hypertension. FEBS Open Bio.

[60] Angeli F, Verdecchia P, Pellegrino C, Pellegrino RG, Pellegrino G, Prosciutti L (2003). Association between periodontal disease and left ventricle mass in essential hypertension. Hypertension.

[61] Santisteban MM, Qi Y, Zubcevic J, Kim S, Yang T, Shenoy V (2017). Hypertension-linked pathophysiological alterations in the gut. Circ Res.

[62] Gomez-Arango LF, Barrett HL, McIntyre HD, Callaway LK, Morrison M, Dekker Nitert M (2016). Increased systolic and diastolic blood pressure is associated with altered gut microbiota composition and butyrate production in early pregnancy. Hypertension.

[63] Toral M, Robles-Vera I, de la Visitacion N, Romero M, Yang T, Sanchez M (2019). Critical role of the interaction gut microbiota - sympathetic nervous system in the regulation of blood pressure. Front Physiol.

[64] Pluznick JL, Protzko RJ, Gevorgyan H, Peterlin Z, Sipos A, Han J (2013). Olfactory receptor responding to gut microbiota-derived signals plays a role in renin secretion and blood pressure regulation. Proc Natl Acad Sci U S A.

